# Supplementary material for: An Algal Metabolite-Based PPAR-γ Agonist Displayed Anti-Inflammatory Effect via Inhibition of the NF-κB Pathway
Source: Mar Drugs. 2019 May 30;17(6):321. doi: 10.3390/md17060321 (PMC6627743; doi:10.3390/md17060321)
Supplement: Supplementary file 1 [file marinedrugs-17-00321-s001.pdf]

An Algal Metabolite-based PPAR-γ Agonist Displayed Anti-Inflammatory Effect via Inhibition of the NF-κB Pathway

Zhiran Ju [1], Mingzhi Su [1], Dandan Li [1], Jongki Hong [2], Dong-Soon Im [1], Suhkmann Kim [3], Eun La Kim [1], and Jee H. Jung [1,*]

[1] College of Pharmacy, Pusan National University, Busan 46241, Republic of Korea

[2] College of Pharmacy, Kyunghee University, Seoul 02447, Republic of Korea

[3] Center for Proteome Biophysics, Department of Chemistry, Pusan National University, Busan 46241, Republic of Korea

* Correspondence: jhjung@pusan.ac.kr (J.H.J.); +82-51-510-2803 (J.H.J.).

List of Figures

Figure S1. The ^{1}H-NMR spectrum of compound (+)-(*R*,*E*)-6a1 3

Figure S2. The ^{13}C-NMR spectrum of compound (+)-(*R*,*E*)-6a1 3

Figure S3. The HRMS data and optical rotation result of compound (+)-(*R*,*E*)-6a1 4

Figure S4. Design of PPAR-γ agonist using algal metabolites, and 15d-PGJ$_2$. 5

Figure S5. *In vitro* PPAR-γ activation by compounds 3l, 6al, 6a2, and by rosiglitazone at 5 µM or 10 µM in rat liver Ac2F cell line 6

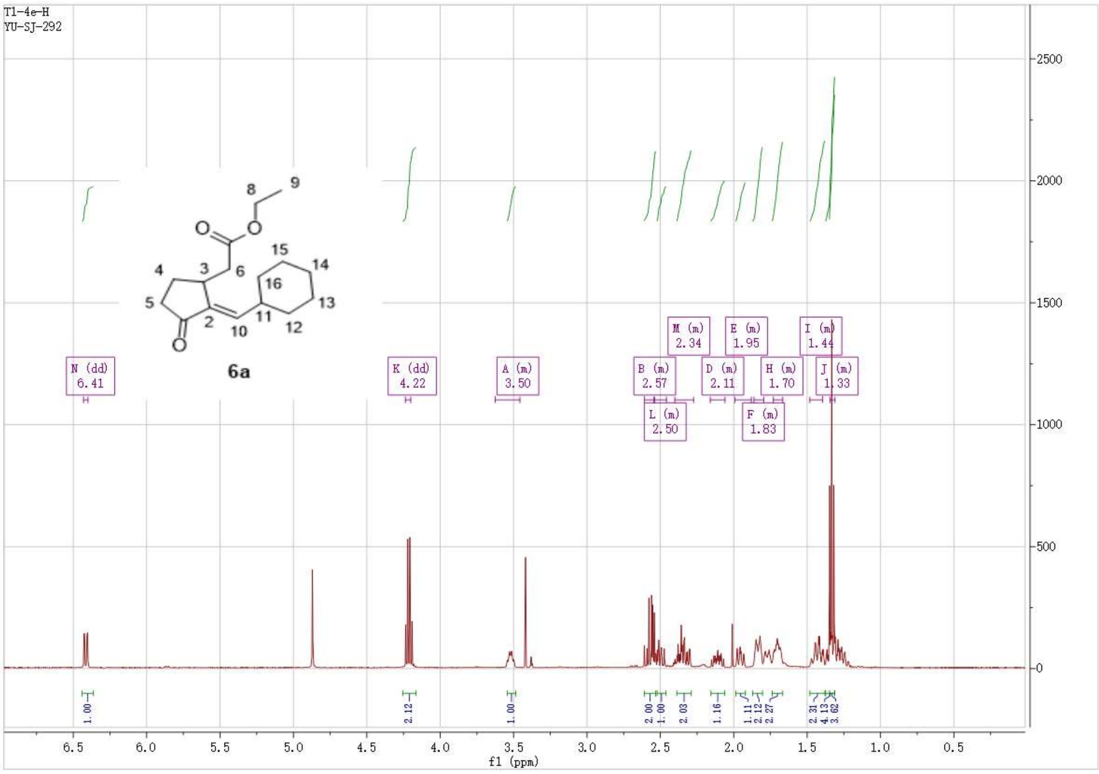

Figure S1. The ^{1}H-NMR spectrum of compound (+)-(*R*,*E*)-6a1

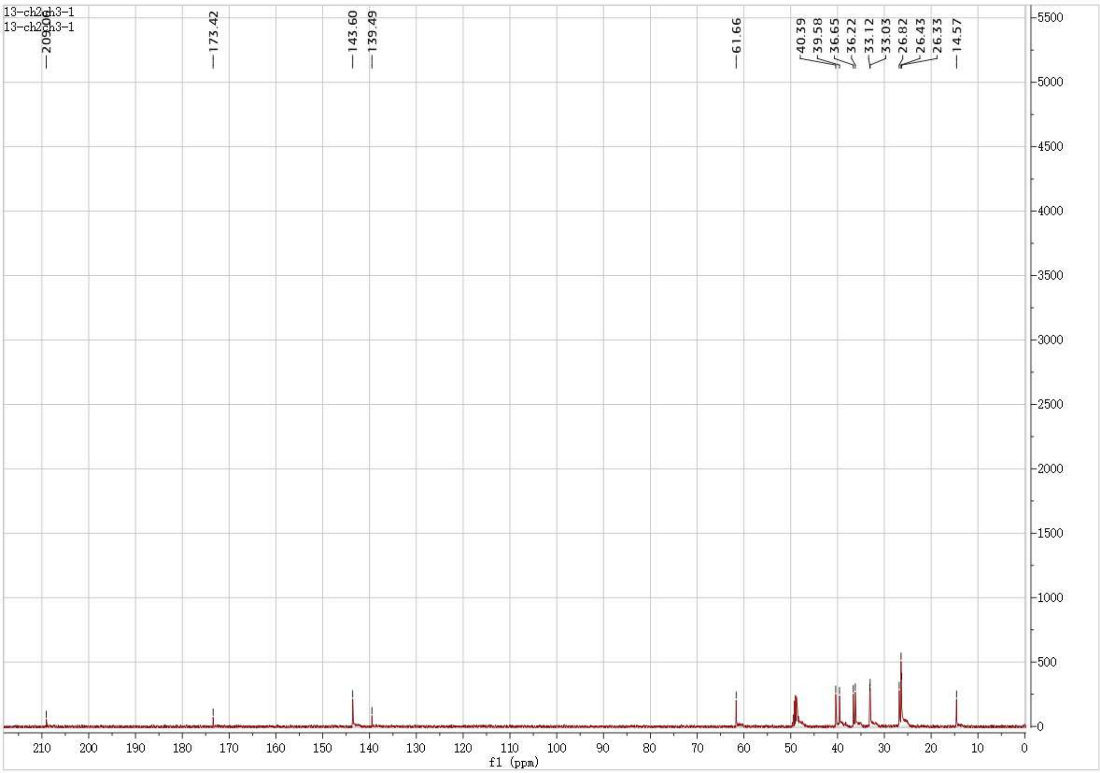

Figure S2. The ^{13}C-NMR spectrum of compound (+)-(*R*,*E*)-6a1

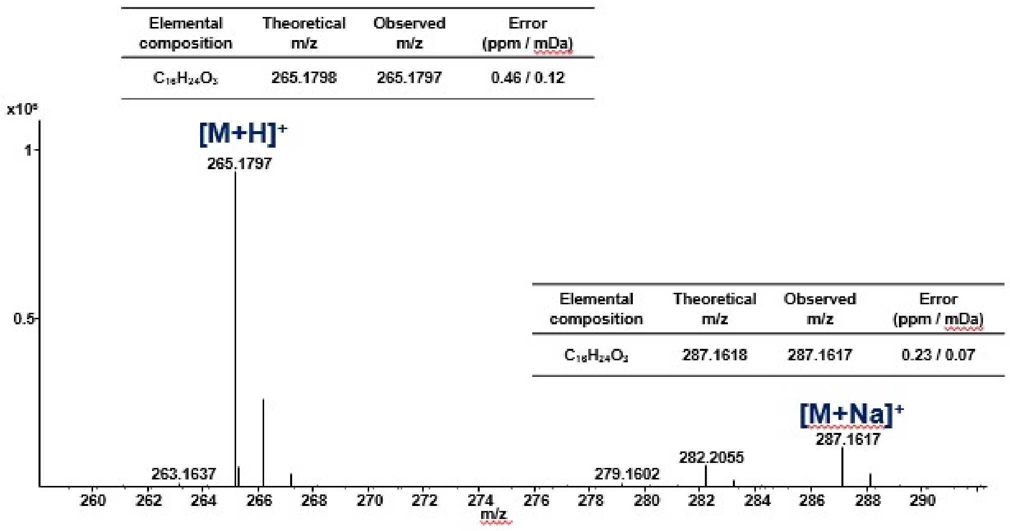

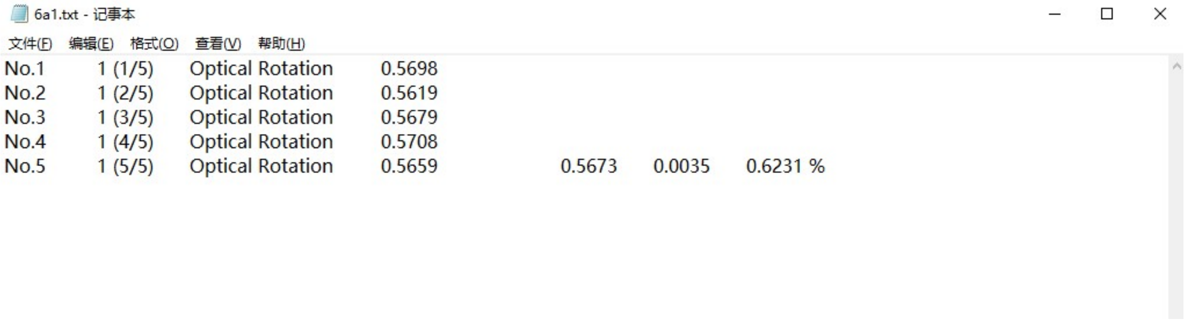
6a1.txt - 记事本
文件(F) 编辑(E) 格式(O) 查看(V) 帮助(H)

No.1	1 (1/5)	Optical Rotation	0.5698			
No.2	1 (2/5)	Optical Rotation	0.5619			
No.3	1 (3/5)	Optical Rotation	0.5679			
No.4	1 (4/5)	Optical Rotation	0.5708			
No.5	1 (5/5)	Optical Rotation	0.5659	0.5673	0.0035	0.6231 %

Figure S3. The HRMS data and optical rotation result of compound (+)-(*R*,*E*)-6a1

HRFABMS *m/z* 265.1797 [M+H]$^+$ (calcd for $C_{16}H_{24}O_3$, 265.1759).

($[\alpha]_D^{20}$ = +5.6, *c* = 0.1, $CHCl_3$)

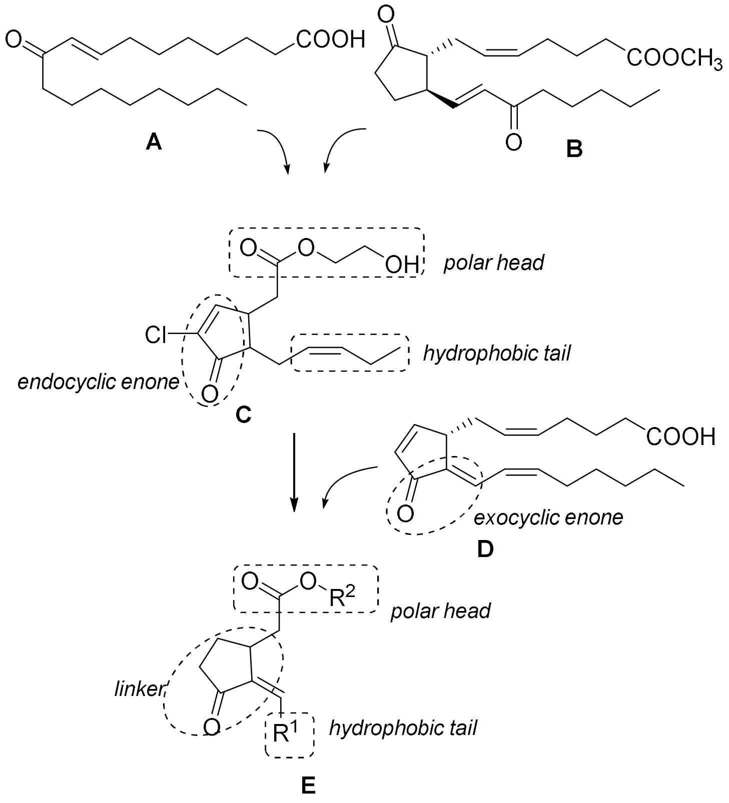

Figure S4. Design of PPAR-γ agonist using algal metabolites, and 15d-PGJ$_2$. (**A**) An oxy fatty acid from the red alga, *Gracilaria verrucosa*. (**B**) A prostaglandin from the red alga, *Gracilaria verrucosa*. (**C**) J11-Cl. (**D**) 15-deoxy-$\Delta^{12,\ 14}$-prostaglandin J$_2$ (15d-PGJ$_2$). (**E**) The designed analogs with an exocyclic enone moiety.

(Ju, Z. R.; Su, M. Z.; Hong J. K.; Ullah, S.; Kim, E. L.; Zhao, C. H.; Moon, H. R.; Kim, S. M. *Eur. J. Med. Chem.* **2018**, *157*, 1192-1201)

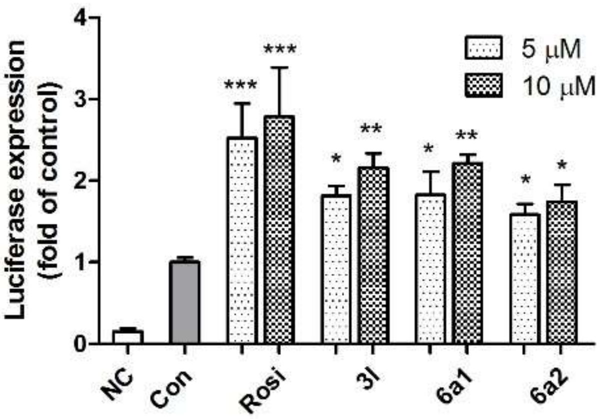

Figure S5. *In vitro* PPAR-γ activation by compounds 3l, 6al, 6a2, and by rosiglitazone at 5 μM or 10 μM in rat liver Ac2F cell line. Cells were transiently transfected with pcDNA or PPRE with pFlag-PPARγ1. NC: negative control, transfected with a plasmid containing PPRE and pcDNA3. Con: control, transfected with a plasmid containing PPRE and pFlag-PPAR-γ1. Rosi: rosiglitazone. Rosiglitazone was used as the positive reference control to monitor the activation of the luciferase reporter. Luciferase expressions (folds of the control) are presented as mean ± SD (n = 3). * $p < 0.05$, ** $p < 0.01$.

(Ju, Z. R.; Su, M. Z.; Hong J. K.; Ullah, S.; Kim, E. L.; Zhao, C. H.; Moon, H. R.; Kim, S. M. *Eur. J. Med. Chem.* **2018**, *157*, 1192-1201)
